# Genetic analysis of iris pigmentation in Swiss pig breeds identifies a missense *KITLG* variant as a potential causal factor for pale and heterochromatic irises

**DOI:** 10.1186/s12711-026-01040-1

**Published:** 2026-03-25

**Authors:** Wim Gorssen, Naveen Kumar Kadri, Negar Khayatzadeh, Alexander S. Leonard, Qiongyu He, Arnav Mehrotra, Stefan Neuenschwander, Hubert Pausch

**Affiliations:** 1 Department of Environmental Systems Science, Animal Genomics, Universitätstrasse 2, 8092 Zurich, Switzerland; 2 SUISAG, Allmend 10, 6204 Sempach, Switzerland; 3 Animal Genetics Unit, Tannenstrasse 1, 8092 Zurich, Switzerland; 4 Present Address: Generatio GmbH, 69126 Heidelberg, Germany

## Abstract

**Background:**

Iris pigmentation is a heritable trait with a complex genetic architecture. While the genetic basis of iris pigmentation has been extensively studied in humans, little is known about iris pigmentation in pigs. Iris pigmentation in pigs varies from different shades of brown or pale irises to heterochromia manifesting either as different colors between both irises (heterochromia iridum) or multiple colors within a single iris (heterochromia iridis). This study investigates the genetics of iris pigmentation variability in the Swiss Landrace and Swiss Large White pig breeds.

**Results:**

Iris pigmentation was phenotyped in 837 Swiss Landrace and 328 Swiss Large White pigs of which the majority also had array-derived genotypes. A high prevalence of heterochromia iridum (18.6%) was observed in the Swiss Landrace breed. Heritability estimates for iris pigmentation were high in both breeds (h^2^ = 57.6–64.4%). Iris pigmentation was not genetically correlated with production traits. Genome-wide association analysis identified several loci associated with iris pigmentation (*P* < 10^–5^), including regions near functional candidate genes such as *TYR, ALX4* and *DCT*. The strongest association was detected near the *KITLG* gene, which was identified as a candidate gene for iris pigmentation in a previous study on Italian Large White pigs. Fine-mapping identified a highly significantly associated (*P* = 2.0 × 10^–12^) missense variant in *KITLG* (5_94084790_G > A, rs342599807, p.R124K) as a potential causal variant for pale and heterochromatic iris pigmentation in Swiss pigs.

**Conclusions:**

Our findings provide new insights into the genetic architecture of iris pigmentation in pigs and indicate that *KITLG* plays a key role. The identification of a putative causal missense variant offers a foundation for further functional studies aiming to better understand pigmentation traits in pigs.

**Supplementary Information:**

The online version contains supplementary material available at 10.1186/s12711-026-01040-1.

## Background

Eye color is determined by pigmentation of the iris, which is a two-layered compact structure composed of connective and smooth muscle tissue that regulates the amount of light entering the eye [1]. Iris pigmentation is determined by four main factors: (i) pigment granules in the posterior pigment epithelium, (ii) pigment concentration in stromal melanocytes, (iii) the type of melanin in these cells (brown-black eumelanin or red-yellow pheomelanin), and (iv) the light-scattering properties of the stromal matrix. Increased pigment in the iris stroma enhances light absorption, resulting in a darker iris pigmentation [1, 2]. High melanin concentrations generally lead to (dark) brown irises, while low melanin concentrations lead to light-colored or pale irises whereas the absence of melanin (e.g. albinism) leads to red or pink irises. Heterochromia is the condition where an individual has two differently colored irises (heterochromia iridum or binocular heterochromia) or variations of color within a single iris (heterochromia iridis or sectorial heterochromia) [1].

Iris pigmentation is a heritable trait with a genetic architecture that is more complex than initially thought [3–7]. Heritability of iris pigmentation is very high in humans; in a twin study, Larsson et al. [8] estimated a broad sense heritability (H^2^) of 85% and a narrow-sense heritability (h^2^) of 51%. Simcoe et al. [7] found 124 independently associated QTL in 61 discrete genomic regions for iris pigmentation in a genome-wide association study (GWAS) involving almost 195,000 individuals.

The *tyrosinase*-encoding gene (*TYR*) has a large impact on iris pigmentation [9] as *tyrosinase* contributes to melanin synthesis, which can later be processed to both eumelanin and pheomelanin [6, 10]. Other genes affecting iris pigmentation in humans are *oculocutaneous albinism II* (*OCA2*) and *HECT and RLD domain containing E3 ubiquitin protein ligase 2* (*HERC2)*, which are known to impact melanosome transport, and *melanocortin 1 receptor* (*MC1R*) which is a regulator of eumelanin and pheomelanin production [6]. Moreover, mutations in the *KIT ligand (KITLG)* gene, which also plays a role in melanogenesis [11], have been linked with skin, hair, and iris pigmentation in humans [12–15]. However, many other genes have been associated with iris pigmentation in humans (e.g., *TYRP*, *DCT*, *SLC24A4*, *SLC45A2*, *IRF4*) [7, 16].

Understanding iris pigmentation in pigs provides insights into the genetic architecture of pigmentation traits. Genes and variants underlying pigmentation traits can have potential implications in breed characterization and might also be associated with other complex defects [4]. However, only few studies have investigated iris pigmentation variability in pigs (Additional File 1 Table S1). Moscatelli et al. [4] reported that 87% of Italian Large White pigs had brown pigmented irises (54.3% dark brown, 14.8% medium brown and 17.9% light brown), 3.8% had pale irises, 5.9% had heterochromia iridum and 3.2% had heterochromia iridis (2.3% unilateral versus 0.9% bilateral). Nielsen and Lind [17] found 50% of Göttingen minipigs had brown irises, 47% had pale irises and 3% had heterochromia (type not specified). Gelatt et al. [3] found 78.5% of miniature pigs had brown irises, while 21.5% had some heterochromia pattern or pale irises. Moreover, they found an association between iris pigmentation and skin and hair color, where pigs with white skin and hair color had higher probability of generating offspring with pale irises or heterochromia, which was previously also observed by Yoshikawa [18]. Yoshikawa reported prevalences of 49.8% brown irises, 14.6% pale irises, 14.7% heterochromia iridum and 20.9% heterochromia iridis [18]. Moscatelli et al. [4] estimated that the h^2^ for heterochromia iridum versus brown irises was between 9.5 and 50.4% for different shades of brown irises in pigs*.* They also found a significant association (*P* < 10^–6^) between several SNPs and iris pigmentation in a GWAS using medium-density microarray-derived genotypes. SNPs near the functional candidate gene *SLC45A2* located on the S*us scrofa* chromosome (SSC) 16 were associated with different shades of brown irises. Pale depigmented irises were associated with SNPs near *EDNRB* (SSC11) and *NOTCH2* (SSC4). Heterochromia iridis was associated with SNPs near *KITLG* (SSC5), *COL17A1* (SSC14) and regions on SSC6 and SSC8. Heterochromia iridum was associated with a region on SSC8.

Here, we investigate this variability in the Swiss Landrace and Swiss Large White pig breeds. First, the prevalence of five iris pigmentation traits is assessed in 1165 pigs. Second, heritabilities of iris pigmentation traits and their genetic correlations with production traits are estimated by integrating performance data, pedigree and genomic information in categorical animal models. Third, GWAS are carried out with haplotypes and imputed sequence variant genotypes to identify trait-associated variants. Last, potentially causal variants are identified in the Swiss Large White population using long and short read sequencing-derived variants.

## Methods

### Animal ethics statement

The pigs described in this study were kept in compliance with the Swiss legislations and were not raised or treated in any way for the purpose of this study.

### Animals and iris pigmentation collection

**Fig. 1:**
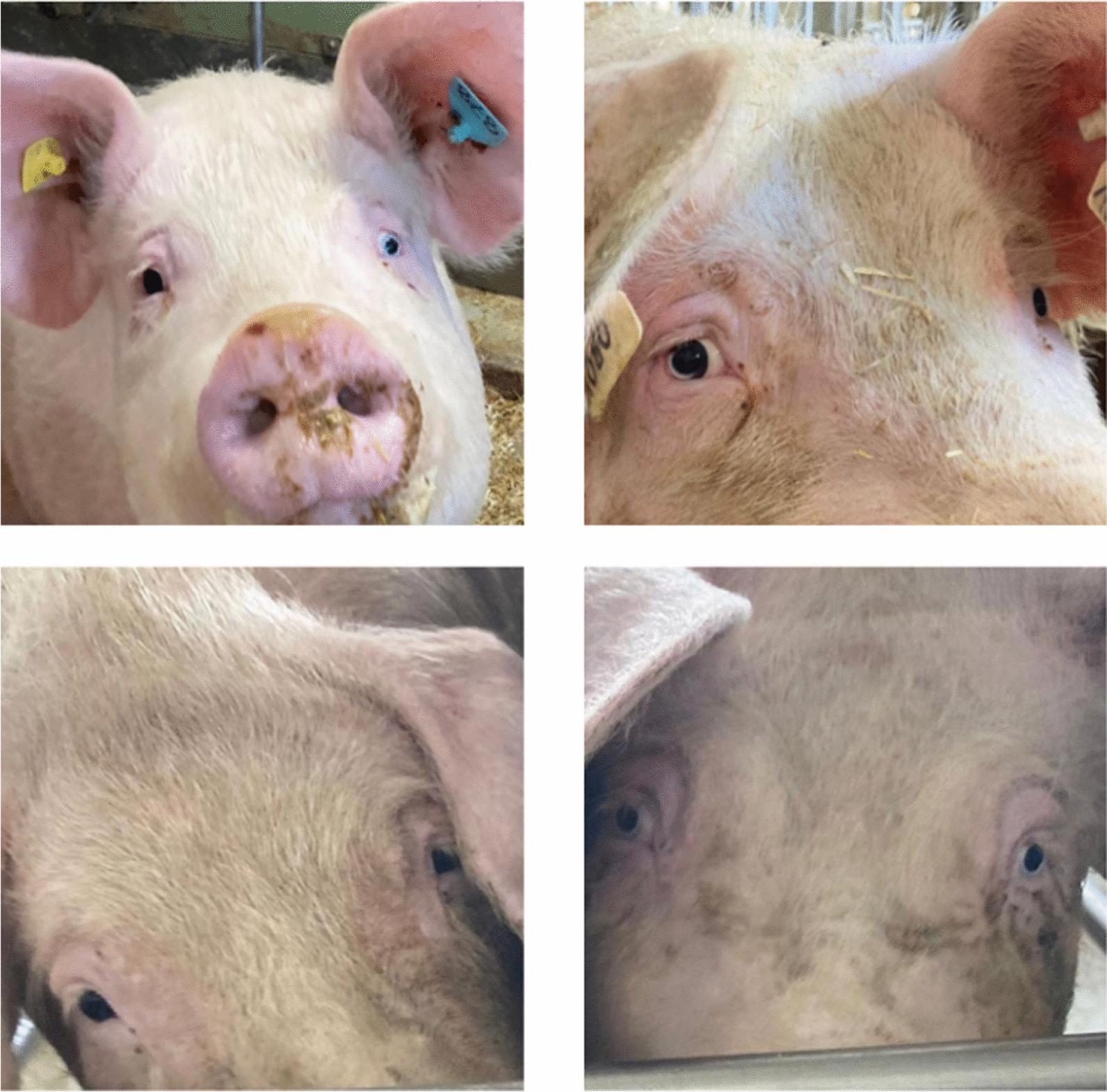
Variability in iris pigmentation observed in Swiss Landrace pigs. Top left: heterochromia iridis, with a brown colored right iris and a pale colored left iris. Top right: Heterochromia iridis unilateralis, where the right iris is both brown and pale. Bottom: two sows with different iris pigmentation: left, a sow with brown pigmented irises, right, a sow with pale pigmented irises

Iris pigmentation was assessed in 2024 in 1210 live pigs at the testing station of the breeding organization SUISAG and at five Swiss pig farms (SUISAG breeders). All pigs that were phenotyped at the SUISAG testing station were at the finishing stage, whereas pigs phenotyped at the farms were either gilts, sows or breeding boars. Iris pigmentation was assessed as described in Moscatelli et al. [4] (Fig. 1, Additional File 2 Fig. S1). Each individual iris was scored as pale or one of three shades of brown: dark, medium or light brown. Differentiating between medium and dark brown irises was difficult under practical conditions with different light intensities, and so these two classes were later considered as ‘dark’. In the case of heterochromia iridis, the dominant iris color was noted first, followed by the secondary color. Some pigs, mainly Swiss Landrace individuals with drooping ears obscuring their eyes, could only be phenotyped for one iris. Animals with incomplete records (e.g., only one iris scored) were not considered for the downstream analyses. The final iris pigmentation dataset included 1165 pigs from the Swiss Landrace (N = 837) and Swiss Large White (N = 328) populations for which records for both irises were available. The Swiss Large White animals descend from either a dam or a sire line, which trace back to the same ancestral population but were bred independently since 2002 [19]. Due to a relatively recent common ancestral population, we consider the Swiss Large White sire and dam line as one population but account for population stratification and relationships in all statistical models (below).

### Phenotype data for production traits

Phenotypes for daily gain (g/d), backfat depth (mm) and loin muscle depth (mm) were available for 611 Swiss Landrace and 283 Swiss Large White pigs. These phenotypes were either recorded on live animals at the testing station of SUISAG or during a field test at the breeder farm. These data were visually inspected for outliers and normality via histograms (Additional File 3 Fig. S2, Additional File 4 Fig. S3), but no outliers were detected or removed.

### Genotype data

Microarray-derived genotypes were provided by SUISAG. The genotype data comprised 77,056 SNPs and 1164 pigs before quality control (757 Swiss Landrace, 407 Swiss Large White). The phenotypic and genotypic datasets overlapped largely, but some animals had only phenotype or only genotype records. The coordinates of the SNPs were determined according to the current porcine reference genome (*Sscrofa11.1*). Reference and alternate alleles were updated accordingly for each SNP and SNPs with duplicated positions were removed. We used PLINK v1.9 [20] to exclude 32 Swiss Landrace and 13 Swiss Large White individuals with call-rate below 95% or an excess of heterozygosity (i.e., deviating more than 3 standard deviations from the mean). All individuals passed the sex check. We excluded SNPs with call rate lower than 95% and minor allele frequency below 1%. For autosomal chromosomes, we also excluded SNPs deviating significantly from Hardy–Weinberg proportions (*P* < 0.0001). For the X chromosome, SNPs in male individuals showing heterozygosity outside the pseudoautosomal region (defined as 0–7 Mbp) were set to missing. The remaining SNPs were then treated as homozygous diploid in subsequent analyses. The SNP-based quality control was performed for each breed separately. The final genotype dataset contained 725 pigs and 58,229 SNPs for Swiss Landrace and 394 pigs and 54,426 SNPs for Swiss Large White. A combined dataset was created based on the overlapping SNPs after per breed QC, containing 1119 pigs and 50,143 SNPs. Of the genotyped individuals, phenotypic records were available for 586 Swiss Landrace pigs, 303 Swiss Large White pigs and 889 pigs in the combined dataset.

After quality control, a principal component analysis (PCA) was performed with PLINK v1.09 (-pca) on the combined dataset to examine population structure (Additional File 5 Fig. S4).

### Phasing and imputation

The array-derived genotypes were phased, and sporadically missing genotypes were imputed for each breed separately using Beagle v5.4 software [21] (beagle.01Mar24.d36.jar). The phased array-derived autosomal genotypes of Swiss Large White pigs were imputed to the sequence level based on an in-house reference panel [22] consisting of 120 Swiss Large White pigs that were sequenced (Illumina short-read) to at least 10 × autosomal coverage (mean = 15.9; sd = 5.0; max = 36.8) and which had genotypes at 31,652,340 autosomal sequence variants. None of the sequenced pigs had iris pigmentation phenotypes. The genetic relationship between the genotyped population and the sequenced reference population was inspected through PCA (Additional File 6 Fig. S5). Reference-based imputation was performed using Beagle with an effective population size of 100, a window of 10, overlap 2, and burn-in 5 [23].

Sequence variant genotypes for the Swiss Landrace pigs were inferred using the SWine IMputation haplotype reference panel (SWIM; https://www.swimgeno.org/) [24]. Sex chromosomes were not imputed, as this was not possible within the SWIM framework.

After imputation, there were 31,652,340 SNPs with a mean r^2^ of 0.710 for Swiss Large White and 34,615,361 SNPs with a mean r^2^ of 0.725 for Swiss Landrace. However, only SNPs with a minor allele frequency > 0.01 and an r^2^ value > 0.5 were retained, leading to 22,378,549 SNPs with mean r^2^ of 0.941 for Swiss Large White and 15,631,491 SNPs with mean r^2^ of 0.935 for Swiss Landrace. For the combined analysis of the Swiss Large White and Swiss Landrace pigs, overlapping SNPs after per-breed QC were retained (12,116,974 SNPs).

### Estimation of genetic parameters

Genetic parameters were estimated with *gibbsf90* + software [25] using a Bayesian method implementing single-step genomic prediction (OPTION SNP_file). A threshold model was used with five categorical phenotypes (OPTION cat 5; dark brown, light brown, pale, heterochromia iridum and heterochromia iridis) for each breed separately. All models were run with 500,000 samples, a burnin of 100,000 and a sample rate of 1000. Mean estimates and 95% highest posterior density intervals (HPD) were reported.

The estimated sire-dam models were of the form:

1 $$ {\mathbf{y}} = {\mathbf{Xb}} + {\mathbf{Za}} + {\mathbf{e}} .$$

In Eq. (1), ${\mathbf{y}}$ is the vector with categorical iris pigmentation phenotypes; ${\mathbf{b}}$ is a vector with fixed effects and covariates. The fixed effects were farm-date (6 levels) and sex (3 levels: sow, boar, barrow); ${\mathbf{a}}$ is a vector containing additive genetic animal effects (2496 animals in the pedigree and 725 with genotype information for Swiss Landrace; 3710 animals in the pedigree and 394 with genotype information for Swiss Large White). The assumption is that ${\mathbf{a}}$ follows a normal distribution for the ${\mathbf{H}}$ matrix following [26–28], using single-step genomic evaluation with both pedigree (${\mathbf{A}}$) and genomic (${\mathbf{G}}$) relationship matrices: ${\mathbf{a}}\sim {\mathrm{N}}\left( {0,{\mathbf{H}}{\upsigma }_{{\mathrm{a}}}^{2} } \right)$. ${\mathbf{e}}$ is the vector of residual effects assumed to follow a normal distribution ${\mathbf{e}}\sim N\left( {0,{\mathbf{I}}\sigma_{e}^{2} } \right)$; ${\mathbf{X}}$ and ${\mathbf{Z}}$ are incidence matrices for respectively fixed effects and random animal effects. The **H** matrix was constructed using the array-derived medium density genotypes.

Next, we estimated genetic correlations (r_g_) of iris pigmentation with the production traits life daily gain (g/day), backfat thickness (mm) and loin muscle depth (mm) using bivariate models. Genetic correlations were only retained for the Swiss Landrace population, as sample size was insufficient to reliably estimate genetic correlations for our Swiss Large White dataset (N = 328). Genetic correlations were estimated as follows:

2 $$ \left[ {\begin{array}{*{20}c} {{\mathbf{y1}}} \\ {{\mathbf{y2}}} \\ \end{array} } \right] = \left[ {\begin{array}{*{20}c} {{\mathbf{X1}}} & {\mathbf{0}} \\ {\mathbf{0}} & {{\mathbf{X2}}} \\ \end{array} } \right]\left[ {\begin{array}{*{20}c} {{\mathbf{b1}}} \\ {{\mathbf{b2}}} \\ \end{array} } \right] + \left[ {\begin{array}{*{20}c} {{\mathbf{Z1}}} & {\mathbf{0}} \\ {\mathbf{0}} & {{\mathbf{Z2}}} \\ \end{array} } \right]\left[ {\begin{array}{*{20}c} {{\mathbf{a1}}} \\ {{\mathbf{a2}}} \\ \end{array} } \right] + \left[ {\begin{array}{*{20}c} {{\mathbf{e1}}} \\ {{\mathbf{e2}}} \\ \end{array} } \right] $$

Similar to the sire-dam model, ${\mathbf{y1}}$ from Eq. (2) represents a vector with categorical iris pigmentation phenotypes with 5 categories and ${\mathbf{y2}}$ represents a vector with one of the continuous production trait phenotypes; ${\mathbf{b1}}$ and ${\mathbf{b2}}$ are vectors containing fixed effects and covariates, which were farm-date of sampling (6 levels), sex (3 levels: sow, boar, barrow) and age at recording of production data (covariate); ${\mathbf{a1}}$ and ${\mathbf{a2}}$ are vectors of additive animal genetic effects, assumed to follow a normal distribution for the ${\mathbf{H}}$ matrix using single-step genomic evaluation:

3 $$ \left[ {\begin{array}{*{20}c} {{\mathbf{a1}}} \\ {{\mathbf{a2}}} \\ \end{array} } \right]\sim {\mathrm{N}}\left( {\left[ {\begin{array}{*{20}c} {\mathbf{0}} \\ {\mathbf{0}} \\ \end{array} } \right],\left[ {\begin{array}{*{20}c} {{\upsigma }_{{{\mathrm{a}}1}}^{2} } & {{\upsigma }_{{{\mathrm{a}}1,{\mathrm{a}}2}} } \\ {{\upsigma }_{{{\mathrm{a}}1,{\mathrm{a}}2}} } & {{\upsigma }_{{{\mathrm{a}}2}}^{2} } \\ \end{array} } \right] \otimes {\mathbf{H}}} \right) $$

${\mathbf{e1}}$ and ${\mathbf{e2}}$ from Eq. (2) are vectors of residual effects, assumed to follow a normal distribution

4 $$ \left[ {\begin{array}{*{20}c} {{\mathbf{e1}}} \\ {{\mathbf{e2}}} \\ \end{array} } \right]\sim {\mathrm{N}}\left( {\left[ {\begin{array}{*{20}c} {\mathbf{0}} \\ {\mathbf{0}} \\ \end{array} } \right],\left[ {\begin{array}{*{20}c} {{\upsigma }_{{{\mathrm{e}}1}}^{2} } & 0 \\ 0 & {{\upsigma }_{{{\mathrm{e}}2}}^{2} } \\ \end{array} } \right]} \right) $$

${\mathbf{X1}}$, ${\mathbf{X2}}$, ${\mathbf{Z1}}$ and ${\mathbf{Z2}}$ are incidence matrices for fixed effects and random animal effects.

### Genome wide association study

Genome wide association studies were conducted separately for the combined dataset and for both breeds at the haplotype level and on the imputed sequence level. Twelve case-control scenarios of contrasting iris pigmentation traits were considered (Table 1). In total, 72 GWAS analyses were performed: twelve scenarios for three populations at the haplotype and the imputed sequence level.

**Table 1:** Case-control contrast scenarios for iris pigmentation used for GWAS analyses

Scenario	Description	Sample size Swiss Landrace	Sample size Swiss Large White
1: Any brown versus pale	Any brown irises versus pale irises	345 versus 47	233 versus 20
2: Any brown versus iridis	Any brown irises versus heterochromia iridis	345 versus 68	233 versus 28
3: Any brown versus iridum	Any brown irises versus heterochromia iridum	345 versus 126	233 versus 22
4: Any brown versus iridis or iridum	Any brown irises versus heterochromia iridis or iridum	345 versus 194	233 versus 50
5: Any brown versus pale or iridis or iridum	Any brown irises versus pale irises or heterochromia iridis or iridum	345 versus 241	233 versus 70
6: Dark brown versus pale	Dark brown irises versus pale irises	243 versus 47	152 versus 20
7: Dark brown versus iridis	Dark brown irises versus heterochromia iridis	243 versus 68	152 versus 28
8: Dark brown versus iridum	Dark brown irises versus heterochromia iridum	243 versus 126	152 versus 22
9: Dark brown versus iridis or iridum	Dark brown irises versus heterochromia iridis or iridum	243 versus 194	152 versus 50
10: Dark brown versus pale or iridis or iridum	Dark brown irises versus pale irises or heterochromia iridis or iridum	243 versus 241	152 versus 70
11: Dark brown versus light brown	Dark brown irises versus light brown irises	243 versus 92	152 versus 79
12: Iridum versus any brown or pale	Heterochromia iridum versus any brown or pale irises	126 versus 392	22 versus 253

The sample size for the combined analysis of Swiss Landrace and Swiss Large White is the sum of the sample sizes of both breeds

We used the GCTA software (gcta-1.94.1) [29] to conduct GWAS with autosomal imputed sequence variants considering the genomic relationship matrix, its first four principal components to account for population stratification and sex as fixed effect (–mlma, –grm, –qcovar, –covar).

Haplotype-based association analysis was conducted using a sliding window of 10 SNPs with an overlap of 5 SNPs, while considering the first twenty principal components and sex as covariables and a minor allele frequency cutoff of 1%. For haplotypes segregating at a frequency between 0.01 and 0.99, difference in its count in case and control groups was tested using Fisher’s exact test. Haplotype-based GWAS was done on variants on autosomal and sex chromosomes.

Manhattan plots were generated with the *manhattan* function of the *qqman* package in R [30] using a genome-wide significance threshold of *P* < 5 × 10^–8^ and a suggestive significance threshold of *P* < 1 × 10^–5^.

### Identification of candidate genes

**Fig. 2:**
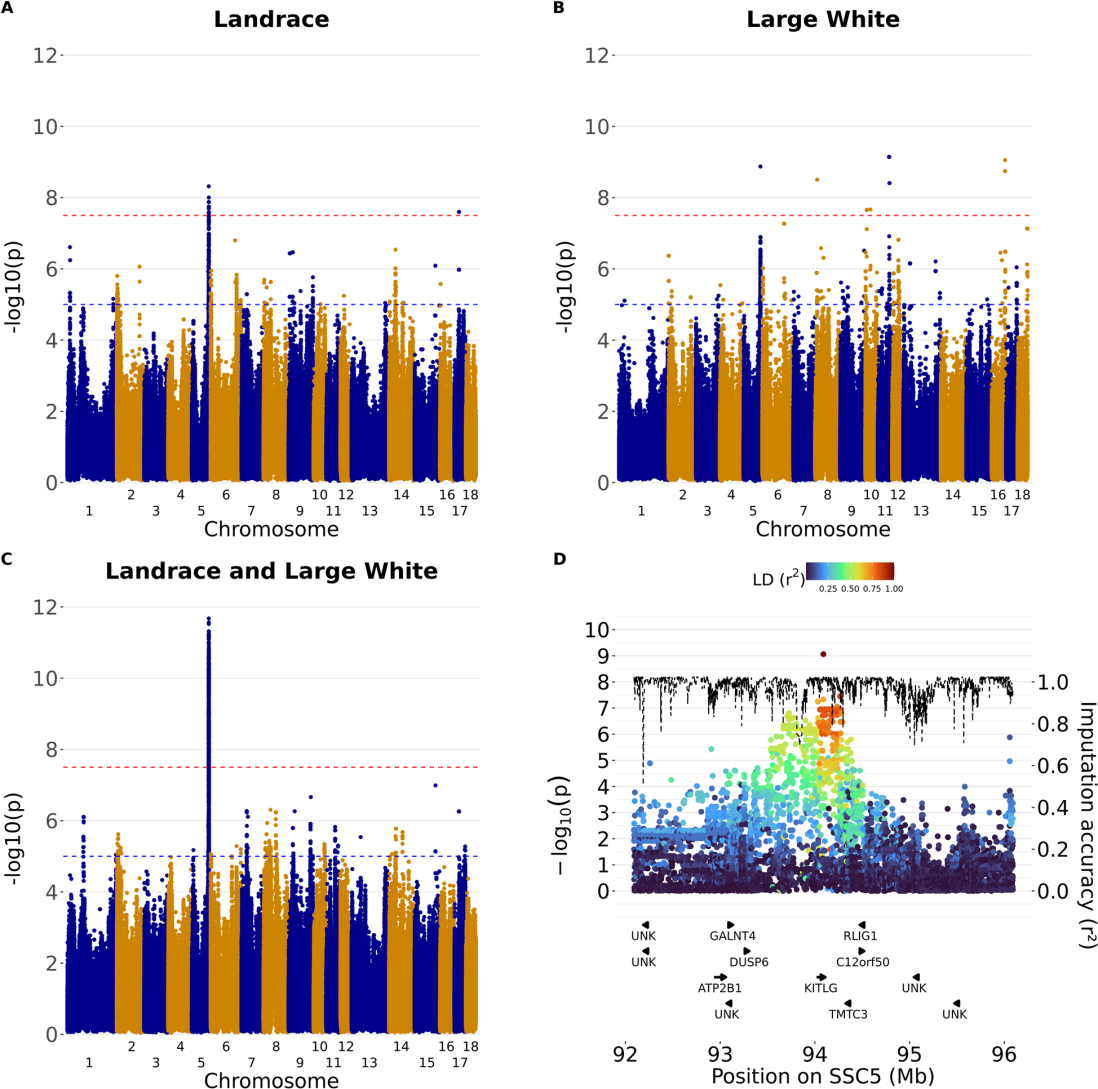
Manhattan plots for iris pigmentation in Swiss pig breeds. For each variant, the most significant association was kept over all twelve GWAS scenarios (Table 1) for Swiss Landrace (**A**), Swiss Large White (**B**), and the combined analysis (**C**). Panel D shows the zoomplot for the association of any brown irises versus pale or heterochromia iridis or iridum (scenario 5, Table 1) in Swiss Large White. The negative logarithm of the p-value is given on the left y-axis, the chromosomal location in Mb is given on the x-axis. For the Manhattan plots, the red horizontal line indicates the genome-wide threshold of *P* < 5 × 10–8, while the blue horizontal line indicates the suggestive threshold of *P* < 1 × 10–5. For the zoomplot, imputation accuracy is given as a dashed line (right y-axis) and the linkage disequilibrium (r^2^) with the top variant is colored according to the legend. Results per GWAS scenario are provided in Additional File S12 Table S7 and Additional File S13 Table S8

Genes annotated in the Sscrofa11.1 genome assembly [31] within a ± 2 Mb region surrounding all significantly associated SNPs and haplotypes were identified by downloading annotated genes using the biomaRt package in R for Ensembl database version 114 [32]. A zoomplot was made via an in-house R-script for genomic regions associated with iris pigmentation, implementing information on linkage disequilibrium and imputation accuracy (example in Fig. 2D). Positional candidate genes were subsequently evaluated for their potential influence on the associated phenotypes based on a comprehensive review of relevant literature.

### Functional annotation of associated variants

Ensembl’s Variant Effect Predictor (VEP, version 114.0) tool [33] was used to predict functional consequences for all variants with *P* < 5 × 10^–8^ in any of the GWAS scenarios (Table 1). The *Sus scrofa* reference genome (*Sscrofa11.1*) and the corresponding Ensembl transcript annotations were considered. Predicted variant effects were categorized according to sequence ontology terms (e.g., synonymous_variant, missense_variant, intron_variant). Variants were also annotated with gene symbols, transcript information, regulatory region consequences, and functional impact scores (SIFT and PolyPhen) where available. For top variants, genotype distribution across breeds worldwide was investigated for 1239 pigs from 43 populations which were online available through the SWIM server platform (https://quantgenet.msu.edu/swim/statistics.php).

### Structural variants near KITLG gene

The presence of structural variants was investigated in the window from 92 to 96 Mb on chromosome 5. Within our sequenced cohort of 120 Swiss Large White pigs, we assessed the haplotype status for the most significantly associated haplotype in this region. We defined three groups: homozygous haplotype carrier, heterozygous haplotype carrier and no haplotype carrier. Using Mosdepth [34], the average normalized (z-score) coverage per 250 bp window was calculated and compared between groups. Windows with an absolute difference > 3 (3 standard deviations from mean) were inspected via Integrative Genomics Viewer (IGV; IGV_2.19.5) [35].

To identify structural variants associated with the haplotype, we also analyzed 18 HiFi-sequenced samples, including 10 Swiss Large White pigs and 8 hybrid animals with a Swiss Large White parent. High molecular weight DNA was extracted from frozen ear or tail tissue or blood, all provided by SUISAG for unrelated studies, using the NEB Monarch® HMW DNA Extraction Kit. Sequencing was performed on the PacBio Sequel IIe platform, with one SMRT cell per sample. HiFi reads were aligned to the same reference genome using pbmm2 v1.17 [36] followed by structural variant calling with sawfish v1.0.1 [37].

## Results

### Iris pigmentation distribution and prevalence per breed

Two thirds (65%) of 837 Swiss Landrace pigs and three quarters (76.5%) of 328 Swiss Large White pigs had brown irises, whereas 7.3% and 6.4% had pale irises (Table 2, Fig. 1). The prevalence of heterochromia iridum differed considerably between breeds (18.6% in Swiss Landrace pigs compared to 7.0% in Swiss Large White pigs). A high prevalence of heterochromia iridum was observed in all six Swiss Landrace farms (minimum 13.6% to maximum 23.4%; Additional File 7 Table S2). Heterochromia iridis had a prevalence of 9.0% in Swiss Landrace versus 10.0% in Swiss Large White.

**Table 2:** Summary of observed iris pigmentation phenotypes in Swiss pig breeds

Iris pigmentation phenotype	Swiss Landrace (N = 837)	Swiss Large White (N = 328)
Dark brown	407 (48.6%)	166 (50.6%)
Light brown	137 (16.4%)	85 (25.9%)
Pale	61 (7.3%)	21 (6.4%)
Heterochromia Iridum	156 (18.6%)	23 (7.0%)
Heterochromia Iridis Unilateralis	64 (7.6%)	28 (8.5%)
Heterochromia Iridis Bilateralis	12 (1.4%)	5 (1.5%)

In total, there were 837 Swiss Landrace pigs and 328 Swiss Large White pigs with an iris pigmentation phenotype. The number of animals with a given iris pigmentation phenotype is shown and the relative percentage within the breed is given between brackets. A detailed overview per farm and per breed is given in Additional File 7 Table S2, whereas an overview per sex is given in Additional File 8 Table S3

A chi-squared test of independence revealed significant differences in iris pigmentation phenotypes between sexes (χ^2^ test, *p* = 0.0004; prevalences in Additional File 8 Table S3). The largest differences between females and males were observed for heterochromia iridum (17.8% vs. 11.4%), light brown (16.6% vs. 23.9%), and pale irises (8.0% vs. 5.3%), respectively.

Sows with dark brown irises had the highest incidence of offspring with dark brown irises (64.1%), whereas sows with pale irises had a low incidence of offspring with dark brown irises (38.2%) (Additional File 9 Table S4). Moreover, sows with heterochromia iridum had the highest incidence of offspring with heterochromia iridum (25.0%).

### Genetic parameters

The h^2^ of iris pigmentation was 57.6% (HPD: 22.5–81.2%) in Swiss Landrace and 64.4% (HPD: 17.3–99.4%) in Swiss Large White (Table 3) on the liability scale. The genetic correlations between iris pigmentation and the three production traits studied had HPD intervals that included zero, indicating no strong evidence for a non-zero correlation.

**Table 3:** Heritability and genetic correlation estimate for iris pigmentation and production traits

Iris pigmentation phenotype	Swiss Landrace	Swiss Large White
Heritability	57.6% (22.5–80.2%)	64.4% (17.3–99.4%)
Genetic correlation life daily gain	0.10 (− 0.32 to 0.50)	–
Genetic correlation backfat depth	− 0.03 (− 0.40 to 0.34)	–
Genetic correlation loin muscle depth	0.00 (− 0.40 to 0.41)	–

Heritability estimates are on the liability scale. The 95% highest posterior density estimates (HPD) are given between brackets. It should be noted that the HPD intervals are large, due to the limited sample size. Genetic correlations were not estimated for Swiss Large White due to insufficient sample size (N = 328). Detailed estimates of variance components are given in Additional File 10 Table S5 and Additional File 11 Table S6

### Genome wide association study for iris pigmentation

Association tests using haplotypes and imputed sequence variants were conducted to identify genomic regions associated with iris pigmentation. In total, 72 association studies were performed using both SNP-based and haplotype-based approaches. The genomic inflation factor (λ) averaged 1.18 (SD = 0.34; range: 0.88–2.19), suggesting that population stratification and related confounding were generally well controlled although inflation was apparent for some association studies. Several regions were significantly associated with iris pigmentation at the genome-wide threshold (*P* < 5 × 10^–8^) across the twelve GWAS scenarios (Table 4, Fig. 2, Additional File 12 Table S7, Additional File 13 Table S8).

**Table 4:** Overview of genetic regions significantly associated (*P* < 5 × 10^–8^) across different GWAS scenarios

Case-control GWAS scenario	Breed	SSC	Top variant or haplotype (bp)	Method	Freq	*P*-value	Candidate gene
Any brown versus pale or iridis or iridum	Landrace	2	12,984,981–13,156,928	Haplotype	0.40	2.3 × 10^–8^	
Any brown versus pale or iridis or iridum	Landrace	5	94,037,661–94238901	Haplotype	0.15	8.5 × 10^–9^	*KITLG*
Any brown versus iridis	Landrace	5	94,178,087	Imputed	0.23	7.2. × 10^–10^	*KITLG*
Any brown versus pale or iridis or iridum	Large White	5	94,089,673	Imputed	0.43	8.7 × 10^–10^	*KITLG*
Any brown versus pale or iridis or iridum	Large White	5	94,040,632–94264923	Haplotype	0.39	7.7 × 10^–9^	*KITLG*
Any brown versus pale or iridis or iridum	Combined	5	94,261,555	Imputed	0.25	1.9 × 10^–13^	*KITLG*
Any brown versus pale	Large White	8	10,799,489	Imputed	0.06	8.2 × 10^–9^	
Any brown versus pale	Large White	10	7,921,953	Imputed	0.06	3.2 × 10^–8^	
Dark brown versus pale	Large White	10	28,724,329	Imputed	0.02	3.4 × 10^–8^	
Iridum versus any brown or pale	Large White	11	62,703,128	Imputed	0.02	3.3 × 10^–10^	*DCT*
Iridum versus any brown or pale	Large White	11	63,954,396	Imputed	0.01	3.0 × 10^–9^	*DCT*
Iridum versus any brown or pale	Large White	11	65,138,862	Imputed	0.01	3.0 × 10^–9^	*DCT*
Any brown versus pale	Large White	16	73,678,712	Imputed	0.02	1.9 × 10^–9^	
Any brown versus iridis	Landrace	17	25,089,995	Imputed	0.01	4.3 × 10^–9^	

The results are ordered by chromosome number and location. Per 1 Mb bin, the most significantly associated SNP or haplotype is shown per breed over all GWAS scenarios. For haplotype-based analyses, no top associated variant was given, as this is uninformative. SSC: Sus scrofa chromosome. Freq: Minor allele frequency or haplotype frequency. Detailed estimates including odds ratios and effect sizes for all significant variants are provided in Additional File 12 Table S7 and Additional File 13 Table S8

Across all combinations tested, the strongest associations between iris pigmentation and genetic markers were found on SSC5 between 94,037,661 and 94,264,923 bp encompassing the *KITLG* gene (Table 4, Fig. 2). This region was identified in both breeds, and in the multi-breed analysis. Significant markers or haplotypes were detected in the case-control scenarios 2, 4, 5, 7, 8, 9, and 10 (see Table 1), in which animals with brown or dark brown eyes are cases, and animals with pale, iridis and/or iridum eyes are controls. Across all GWAS conducted, the most significant association was detected in the imputed SNP-based analysis of scenario 5 (Chr5:94,261,555; *P* = 1.9 × 10^–13^).

Significant associations other than those within the interval from 92 to 96 Mb on SSC5 were only found within but not across breeds. Significant trait-variant associations were observed in the Swiss Landrace population on SSC2 and SSC17 (Table 4), but with no clear underlying functional genes. In the Swiss Large White population, we identified significant associations on SSC8, SSC10, SSC11 and SSC16. Here, the associations detected on SSC11 between 62,703,128 and 65,138,862 bp encompass the functional candidate gene *DCT*.

An overview of the 268 unique haplotypes and 15,419 unique SNPs surpassing the suggestive threshold (*P* < 1 × 10^–5^) is given in both Additional File 12 Table S7 and Additional File 13 Table S8. Although these associations are not all significant on the genome-wide threshold and may contain false-positives, manual inspection of annotated genes surrounding the top variants was conducted. Upon comparing associations across the Swiss Landrace and Swiss Large White populations, overlapping regions with associations (*P* < 1 × 10^–5^) are independently present in both breeds and in the combined analysis on SSC2 (7.6–7.8 Mb), SSC5 (94.1–94.3 Mb; discussed above), SSC7 (29.0–29.4 Mb) and SSC15 (114.5–115.3 Mb). Moreover, several suggestive associations were nearby functional candidate genes. A sequence variant (rs333452789, Chr9:22,593,530) was suggestively associated (*P* = 9.9 × 10^–7^) in scenario 1 in the Swiss Landrace population. This variant is in an intron of the functional candidate gene *TYR*. On SSC8, associations were detected in the region of 40.7–41.1 Mb encompassing the *KIT* gene for scenarios 5, 9 and 10. In this region near *KIT*, the strongest association was found for an imputed sequence variant within the Swiss Landrace population for scenario 10 (Chr8:40,778,203; *P* = 1.2 × 10^–6^). Moreover, in Landrace, suggestive associations were found on SSC2 for scenarios 1, 3, 5, 6 and 10 from 17.8 to 18.8 Mb encompassing the functional candidate gene *ALX4*. Within these regions, the most significant association was found for the haplotype analysis for scenario 5 (Chr2:17,792,133–18,078,400; *P* = 3.3 × 10^–7^).

### Functional annotation of variants associated with iris pigmentation

To functionally annotate variants associated with iris pigmentation, we used Ensembl’s VEP on 5105 unique variants that were associated (*P* < 5 × 10^−8^) in at least one case-control scenario (Additional File 14 Table S9). Among them, 95.9% were known variants, and 4.1% were novel. A total of 16 genes and 42 regulatory features were overlapped by these variants. Most annotated variants were in intronic (44%) and intergenic (40%) regions, followed by downstream (6%) and upstream (4%) gene regions.

Only a small proportion of the variants affected coding regions, with 20 synonymous variants and 18 missense variants. Moderate-impact coding variants (missense) were identified in *KITLG*, *CEP290* and *RLIG1*. From the variants in coding regions, the missense variant in *KITLG* had the strongest association (5_94084790_G > A; rs342599807, KITLG:p.R124K; most significant association of *P* = 2.0 × 10^–12^ for scenario 5 in the combined analysis and imputation accuracy r^2^ = 1). Genotype frequencies of this variant across 43 pig breeds worldwide are provided in Additional File 15 Table S10.

### Structural variants near KITLG gene

We examined the interval from 92 to 96 Mb on SSC5 encompassing *KITLG* for the presence of structural variants in Swiss Large White pigs, as several strongly associated haplotypes and variants within this interval indicate that this region has an impact on iris pigmentation in that population. We focused on the haplotype which was most strongly associated (*P* = 7.7 × 10^–9^; Table 4) with iris pigmentation in the genotyped Swiss Large White pigs. This haplotype consists of 10 consecutive SNPs and spans from 94,040,632 to 94,264,923 bp, thereby partly overlapping the *KITLG* gene (Additional File 12 Table S7, Additional File 13 Table S8). This haplotype occurs at a frequency of 21.2% in the sequenced cohort of 120 Swiss Large White individuals and is also in perfect linkage disequilibrium (r = 1.00) with the *KITLG* missense variant 5_94084790_G > A. Our association tests indicate that haplotype carriers have an increased prevalence of pale or heterochromatic irises. Within the sequenced cohort, there were 6 homozygous haplotype carriers, 39 heterozygous carriers, and 75 non-carriers. We identified 8 regions for which the normalized sequence coverage differed by a factor of at least 3 between homozygous haplotype carriers and non-carriers possibly indicating the presence of deletions or duplications (Chr5:93,895,250–93,895,500; Chr5:93,956,750–93,959,250; Chr5:94,015,000–94,015,250; Chr5:94,036,000–94,036,250; Chr5:94,046,750–94,047,250; Chr5:94,282,250–94,282,500; Chr5:94,286,000–94,286,250; Chr5:94,307,750–94,308,000). For all these regions, the normalized sequence coverage was low for homozygous haplotype carriers, intermediate for heterozygous carriers and normal for non-carriers. Moreover, through manual inspection, we identified a region within the *KITLG* gene near an exon with a normalized coverage difference of 1.14 (Chr5:94,081,500–94,081,750; Additional File 16 Fig. S6). A detailed view of this region in Integrative Genomics Viewer (Additional File 17 Fig. S7) indicates a potential insertion at this location, linked to the haplotype we identified.

A more exhaustive analysis of structural variants was conducted in eighteen pigs for which HiFi long-read sequencing data were available. From these eighteen pigs, six were heterozygous carriers and one was homozygous for the alternate allele of the missense variant 5_94084790_G > A. An alignment-based approach identified 445 structural variants in the interval from 92 to 96 Mb on SSC5 including sixteen that were in high LD (r^2^ > 0.8) with the missense variant 5_94084790_G > A (Additional File 18 Fig. S8 and Additional File 19 Table S11). There were no SVs overlapping coding sequence, but we identified a 316 bp insertion at Chr5:94,081,687 bp in the third intron of *KITLG* (317 bp upstream of exon 4; Additional File 18 Fig. S8 and Additional File 19 Table S11). This insertion matches the repeat annotation for the porcine repetitive element 1 (PRE1), a short interspersed nuclear element (SINE) [38]. This insertion at Chr5:94,081,687 bp and the deletion at Chr5: 94,051,620 bp also coincides with the region detected by manually scanning the *KITLG* region in IGV for the 120 short-read sequenced individuals (Additional File 17 Fig. S7). There was also a 288 bp deletion at Chr5:94,014,953, 2.4 Kb upstream of the first *KITLG* exon (Additional File 19 Table S11, Additional File 18 Fig. S8). This deletion removes a putative Pre0_SS element, which is also a SINE element of the PRE1 family [39] and is just outside a region with a normalized coverage difference greater than three in the short-read data (Chr5:94,015,000–94,015,250; Additional File 16 Fig. S6). The 316 bp insertion at Chr5:94,081,687 bp and the 288 bp deletion at Chr5:94,014,953 were in perfect LD with the missense variant 5_94084790_G > A.

## Discussion

In this study, we investigated the prevalence and genetics of iris pigmentation in the Swiss Landrace and Swiss Large White pig populations. We found a remarkably high prevalence for heterochromia iridum (18.6%) in the Swiss Landrace breed. Iris pigmentation was highly heritable (h^2^ = 57.6–64.4%) and it was not correlated with production traits. Our GWAS identified multiple genome-wide significant associations (*P* < 5 × 10^–8^) near candidate genes such as *DCT* and *KITLG*. Fine-mapping identified a missense variant (5_94084790_G > A) as a plausible candidate causative variant within *KITLG* for pale and heterochromatic iris pigmentation in Swiss pigs. However, further functional investigations are needed to establish causality of the missense variant or other candidate causal variants.

The observed prevalences of iris pigmentation phenotypes in the Swiss breeds were comparable to those reported previously in other populations (Additional File 1 Table S1). However, direct comparisons need to be interpreted with caution due to the limited number of available studies and substantial differences in phenotyping methods, populations examined, and time periods. Iris pigmentation variability in the Swiss Large White population was comparable to that reported in Italian Large White pigs [4], phenotyped with a similar methodology. Nonetheless, we observed a somewhat higher prevalence of pale irises (6.4% vs 3.8%), heterochromia iridum (7.0% vs 5.9%), and heterochromia iridis (10.0% vs 3.2%). The most striking observation was the 18.6% prevalence of heterochromia iridum in Swiss Landrace pigs, which is higher than previously reported in other, non-Landrace populations (Additional File 1 Table S1). Heterochromia iridum is very rare (< 1%) in humans [40]. Our findings of a high inter-breed variability of heterochromia iridum in pigs is in line with observations in dogs and cats, where this trait has remarkably high prevalences in some breeds (e.g., Siberian Husky, Border Collie) but may be much rarer in others [41]. As cats and dogs are companion animals, they may have been actively selected for iris pigmentation, such as pale or heterochromatic irises [42]. However, active selection for specific iris pigmentation phenotypes seems unlikely in Swiss Landrace pigs, as iris pigmentation is often obscured by their overhanging ears and no significant genetic correlations with major production traits were identified. Instead, the high incidence of heterochromia may be explained by genetic drift and/or genetic hitchhiking linked to other (re)productive traits not examined in this study. Notably, we found significant differences in iris pigmentation across sexes, with females having more heterochromia iridum than males (17.8% versus 11.4%). In humans, Stelzer [43] also reported an increased prevalence of heterochromia in females (0.37% versus 0.16%), although this study has been criticized for only considering people from Vienna [40]. Other studies also indicated sexual dimorphism in iris pigmentation in humans [44, 45], but not specific to heterochromia. Although we observed sexual dimorphism, no X-chromosome variants surpassed the genome-wide significance threshold, but some X-chromosome haplotypes did surpass the suggestive threshold (Additional File 12 Table S7).

Iris pigmentation is highly heritable (h^2^ = 57.6–64.4%) on the liability scale in the two pig populations studied. While this is consistent with heritability estimates in humans (h^2^ = 51%, H^2^ = 85%) [8], our estimates are higher than those reported previously in other pig populations (h^2^ = 9.5–50.4%) [4]. This discrepancy may be partly due to differences in the statistical approaches used to estimate heritability. We employed a categorical threshold model that integrated both pedigree and genomic information. This method might be better to account for additive genetic effects and familial structure, whereas Moscatelli et al. [4] relied solely on SNP-based data analyzed with the GEMMA software under various iris pigmentation scenarios.

Our GWAS yielded several associations that were significant at the genome-wide significance threshold (*P* < 5 × 10^–8^). The strongest associations across breeds were found on SSC5 near the gene *KIT ligand* (*KITLG;* Chr5:94,017,387–94,110,214). The *KITLG* gene is a pleiotropic cytokine that binds the KIT receptor to regulate diverse processes including melanogenesis, hematopoiesis, stem cell maintenance, and cell migration, acting through pathways like PI3K-AKT, MAPK, and STAT signaling [11]. *KITLG* has been associated with pigmentation traits in several species, such as pigs [4, 5], cattle [46], goats [47], and humans [13–15, 48].

Our fine-mapping efforts prioritized a missense variant (5_94084790_G > A) in the *KITLG* gene as a candidate causal variant for pale and heterochromatic irises in pigs. The prioritized missense variant is close to the top variant reported by Moscatelli et al. [4] (Chr5:94,284,630; *P* = 1.0 × 10^–11^). Analysis of 1,239 pigs from 43 breeds revealed a pronounced population-structure signal in the missense variant (Additional File 15 Table S10). European commercial breeds and European wild boar were almost fixed for the reference (G) allele, whereas many Asian breeds were fixed or nearly fixed for the alternate (A) allele.

Several *KITLG* missense variants affecting the *KIT* binding domain have been identified previously to underly pigmentation disorders in humans [12, 48] including a loss-of-function variant (p.Leu104Val) which also causes heterochromia iridis and progressive hearing loss [15]. The missense variant associated with porcine eye pigmentation also resides in the KIT binding domain of *KITLG*. Although the Swiss pig breeds from this study have a dominant white coat, they have some variability in skin pigmentation as described earlier in both Landrace and Large White breeds [49]. However, we did not score skin hypo- or hyperpigmentation in this study and hearing ability was also not evaluated. Behavioral abnormalities that could possibly result from impaired hearing were not reported by the farmers. It is therefore likely that the prioritized missense variant is not a loss-of-function allele which agrees with a SIFT score of 1 (range of 0–1, where 1 is benign and 0 is deleterious) which suggests it is benign for protein function.

Several small and structural variants are in LD with the KITLG missense variant (Additional File 19 Table S11). We detected a 316 bp insertion (Chr5:94,081,687) in high LD with the missense variant (r^2^ > 0.9; Additional File 18 Fig. S8). This insertion is located 317 bp upstream of the fourth exon of KITLG and matches the annotation for the porcine repetitive element 1 (PRE1). PRE1 elements are the most abundant SINEs in the pig genome, and differences in their methylation have been reported to influence promoter and enhancer activity of adjacent genes [39, 50] The missense variant was also in high LD (r^2^ > 0.8) with a deletion matching a Pre0_SS element, another SINE of the PRE1 family [39], located 2.4 kb upstream (Chr5:94,014,953) of KITLG. A possible impact of structural variations on expression and function of *KITLG* has been described previously: in dogs a 6 kb copy number variant upstream of *KITLG* is associated with coat pigment intensity [51], and in mice a regulatory enhancer of *KITLG* is shown to influence hair pigmentation [52]. While these structural variants overlap putative regulatory elements, the missense variant is also a compelling functional candidate gene as it alters the KIT-binding domain of *KITLG* and is supported by multiple examples where coding changes impact pigmentation phenotypes [15, 48, 51, 52]. Further research involving functional data is required to establish causal links between the missense variant and any other highly significantly associated variants identified nearby and iris pigmentation.

Our GWAS also identified several associations surpassing the suggestive threshold (*P* < 10^–5^, Additional File 12 Table S7) nearby genes that have been implicated to underly eye, skin, or coat color variation in other species. The close vicinity of functional candidate genes adds confidence that some of the suggestive associations are true positives. For instance, we found suggestive associations nearby *KIT*, *DCT*, *ALX4*, *SFMBT2* and *TYR* [5, 7, 9, 41, 53–55]. The associations in the Swiss Landrace and Swiss Large White breeds near *ALX4 (*Chr2:18,037,401–18,085,683) are noteworthy considering that variants nearby *ALX4* were strongly associated with pale irises and heterochromia iridum in Siberian Huskies [41].

This study presents novel insights into the genetic basis of iris pigmentation in pigs. However, several limitations should be acknowledged. First, phenotyping was conducted under practical field conditions on live animals, which may have introduced inaccuracies, particularly in Swiss Landrace pigs where overhanging ears often obscure the eyes. This may have led to under- or misclassification of iris pigmentation phenotypes, especially subtle variations such as light versus medium brown irises. Future studies could benefit from (post-mortem) assessment using standardized tools such as reflectance spectrometry or high-resolution imaging to improve accuracy and reproducibility of pigmentation scoring. Second, this study only included pig breeds with a dominant white coat color. As previous studies have shown that coat color can be associated with iris pigmentation [3, 18] investigating the genetics of iris pigmentation in breeds with different coat colors would be valuable. Although outside the scope of this study, anecdotal observations of pale irises and heterochromia in Piétrain pigs, a spotted breed, further support the potential relevance of studying iris pigmentation variation in non-white breeds. Third, although whole-genome sequence data enabled the detection of promising candidate variants and structural changes near the *KITLG* gene, these findings were not directly supported by animals with phenotypic records and sequence data. This limitation restricts the ability to definitively link specific mutations to observed phenotypes. Expanding the dataset to include phenotyped individuals with matched high-resolution genotypes or sequence information would enhance the power to confirm causal variants. Fourth, the quality of SNP imputation may have been suboptimal, particularly for the Swiss Landrace population, which was imputed using the publicly available SWIM tool [24]. Although the SWIM tool contains 651 Landrace animals in the reference panel, these pigs come from different populations than the Swiss Landrace pigs. These differences in population structure or reference panel composition may have impacted imputation accuracy, potentially affected downstream association results, and limited power. Fifth, this study did not include functional validation of the identified candidate variants near *KITLG*. Without experimental follow-up, these variants remain putative. While phenotyping and data limitations warrant cautious interpretation, this work provides a strong foundation for future functional studies on pigmentation traits in pigs.

## Conclusion

The present study provides new insights into the genetic architecture of iris pigmentation in pigs. We report a remarkably high prevalence of heterochromia iridum in Swiss Landrace pigs (18.6%) and demonstrate that iris pigmentation is a highly heritable trait (h^2^ = 57.6–64.4%) that is genetically not significantly correlated to production traits. Through genome-wide association analyses, we identified several candidate loci associated with iris pigmentation, with the strongest and most consistent signals near the *KITLG* gene on SSC5. Our data support the involvement of a missense variant (5_94084790_G > A) as potential causal mutation underlying pale and heterochromatic irises. Moreover, several structural variants, such as a PRE1 insertion, are in high linkage disequilibrium with this missense variant in Swiss Large White pigs. Additional associations *(P* < 10^–5^) near genes such as *DCT*, *ALX4*, *TYR* and *KIT* suggest a polygenic inheritance of porcine eye colour involving multiple pigmentation-related pathways. While phenotyping and data limitations warrant cautious interpretation, this work lays a strong foundation for future functional studies on pigmentation traits in pigs.

## Supplementary Information

Additional file1 (XLSX 10 KB)

Additional file2 (PNG 366 KB)

Additional file3 (PNG 70 KB)

Additional file4 (PNG 68 KB)

Additional file5 (PNG 505 KB)

Additional file6 (PNG 495 KB)

Additional file7 (XLSX 10 KB)

Additional file8 (XLSX 11 KB)

Additional file9 (XLSX 10 KB)

Additional file10 (XLSX 11 KB)

Additional file11 (XLSX 17 KB)

Additional file12 (XLSX 57 KB)

Additional file13 (XLSX 5167 KB)

Additional file14 (XLSX 1247 KB)

Additional file15 (XLSX 14 KB)

Additional file16 (PNG 521 KB)

Additional file17 (PNG 336 KB)

Additional file18 (PDF 74 KB)

Additional file19 (XLSX 9 KB)

Additional file20 (DOCX 17 KB)

## Data Availability

Genotyping data were provided by SUISAG. The datasets generated and/or analyzed during the current study are not publicly available due to restrictions from SUISAG. High-coverage sequencing read data of the 120 Large White pigs used in this study are available at the European Nucleotide Archive (ENA) (http://www.ebi.ac.uk/ena) of the EMBL at BioProject PRJEB38156 and PRJEB39374. Sequence information to estimate genotype frequency for the missense variant for 1239 pigs across 43 breeds were downloaded via: https://quantgenet.msu.edu/swim/statistics.php.
