# Supplementary figures and images for: Genetic analysis of iris pigmentation in Swiss pig breeds identifies a missense KITLG variant as a potential causal factor for pale and heterochromatic irises
Source: Genet Sel Evol. 2026 Mar 25;58:22. doi: 10.1186/s12711-026-01040-1 (PMC13023151; doi:10.1186/s12711-026-01040-1)

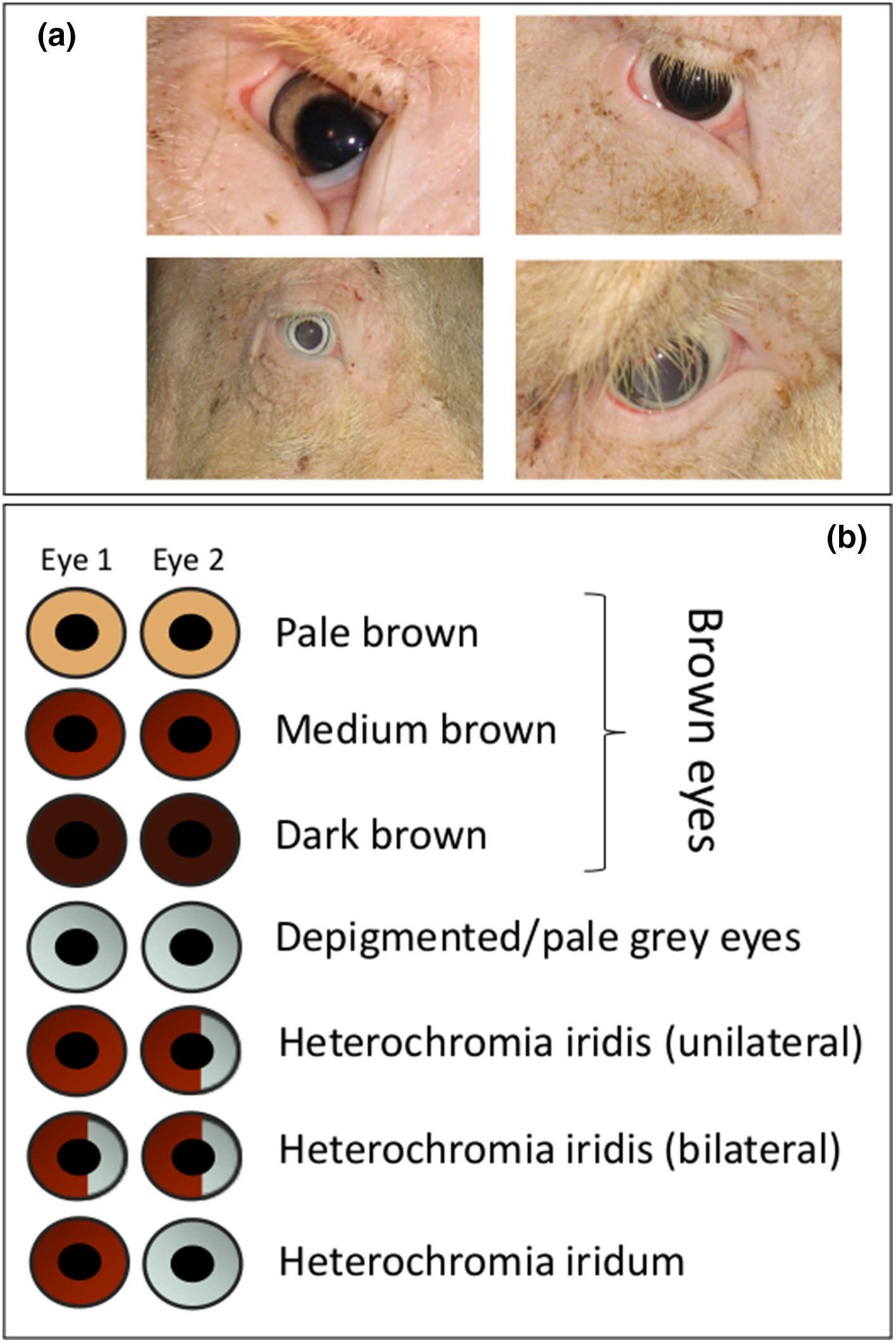

Supplement: Supplementary file 2 — Additional file2 (PNG 366 KB) [file 12711_2026_1040_MOESM2_ESM.png]

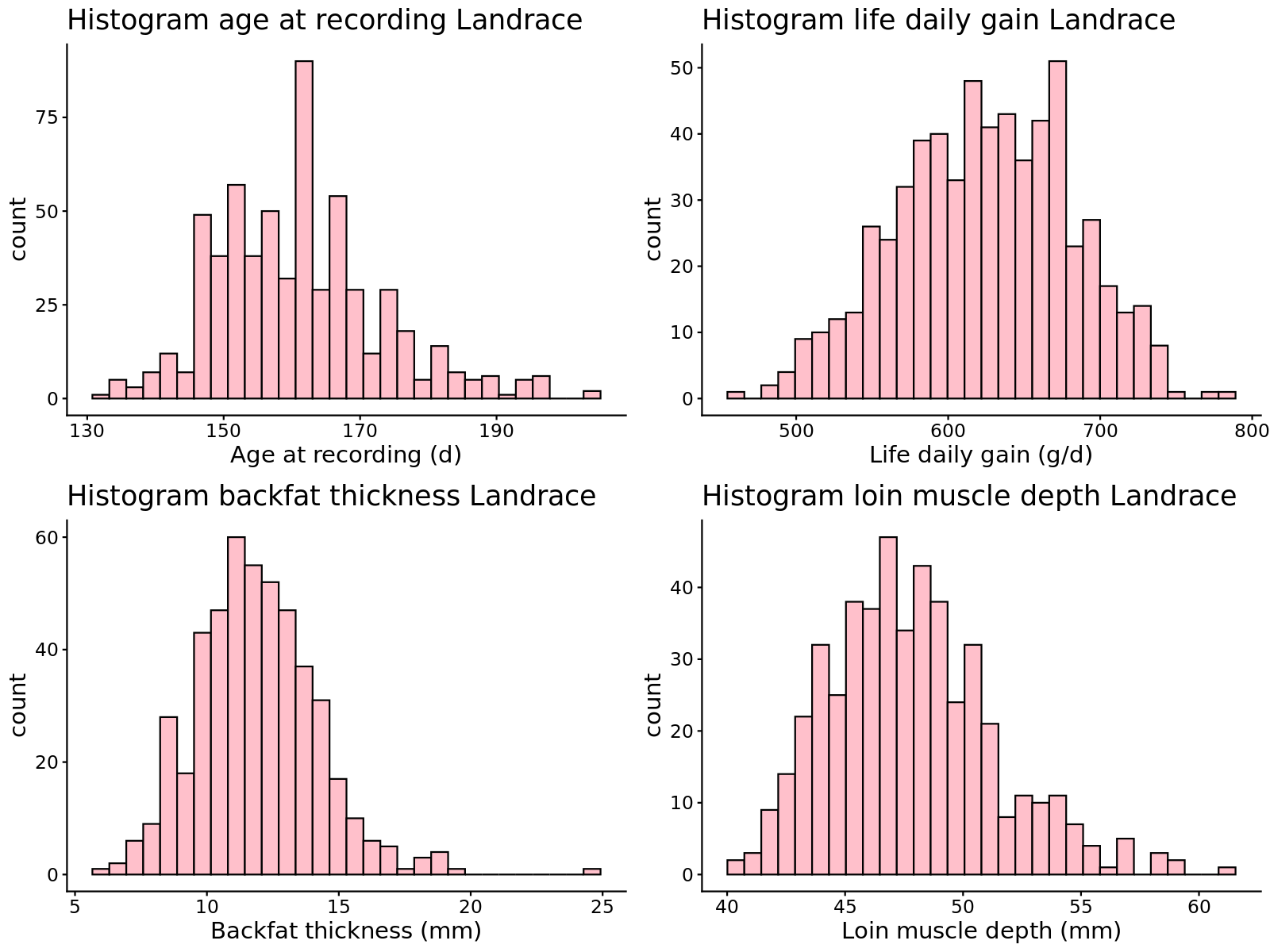

Supplement: Supplementary file 3 — Additional file3 (PNG 70 KB) [file 12711_2026_1040_MOESM3_ESM.png]

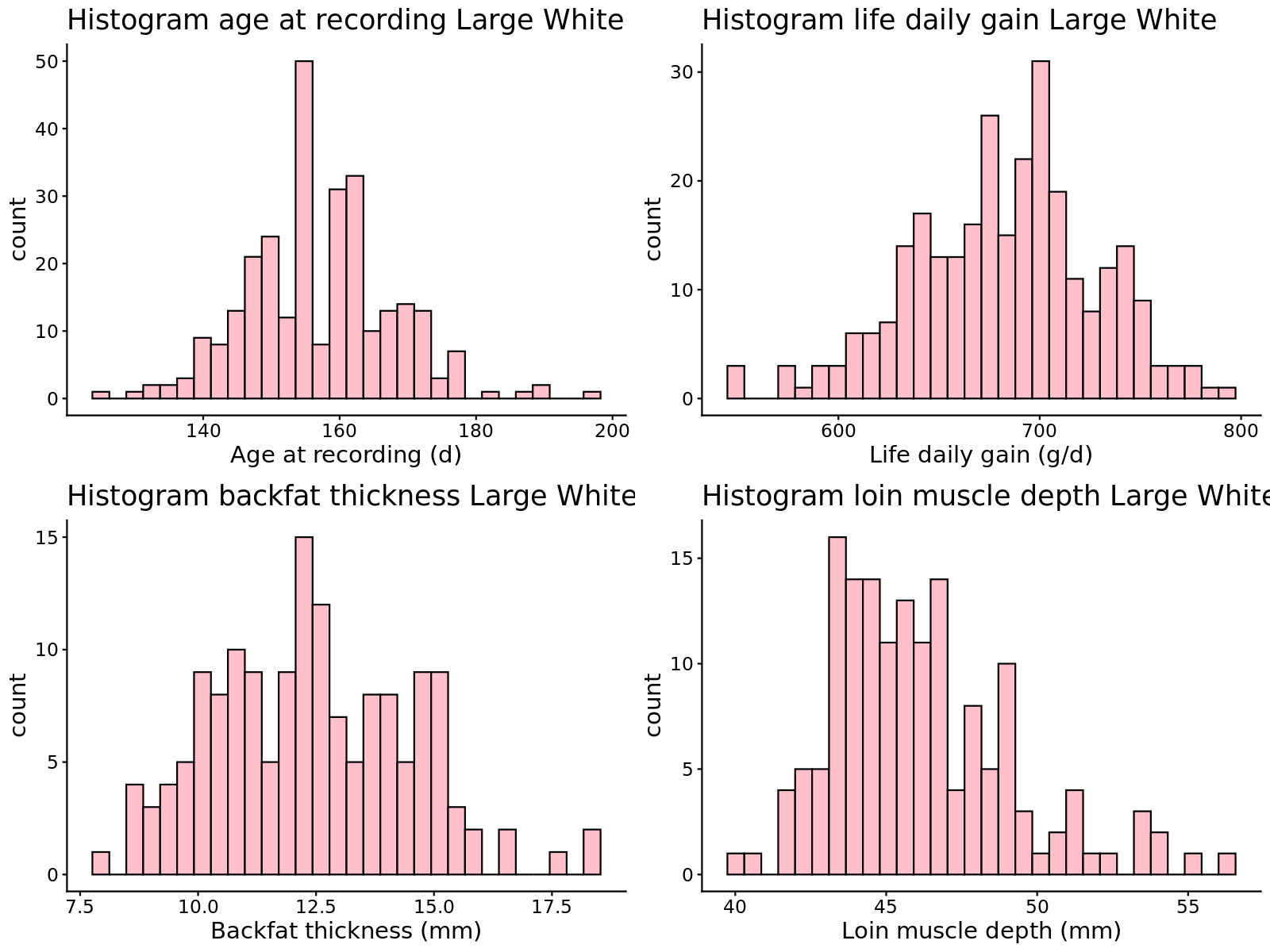

Supplement: Supplementary file 4 — Additional file4 (PNG 68 KB) [file 12711_2026_1040_MOESM4_ESM.png]

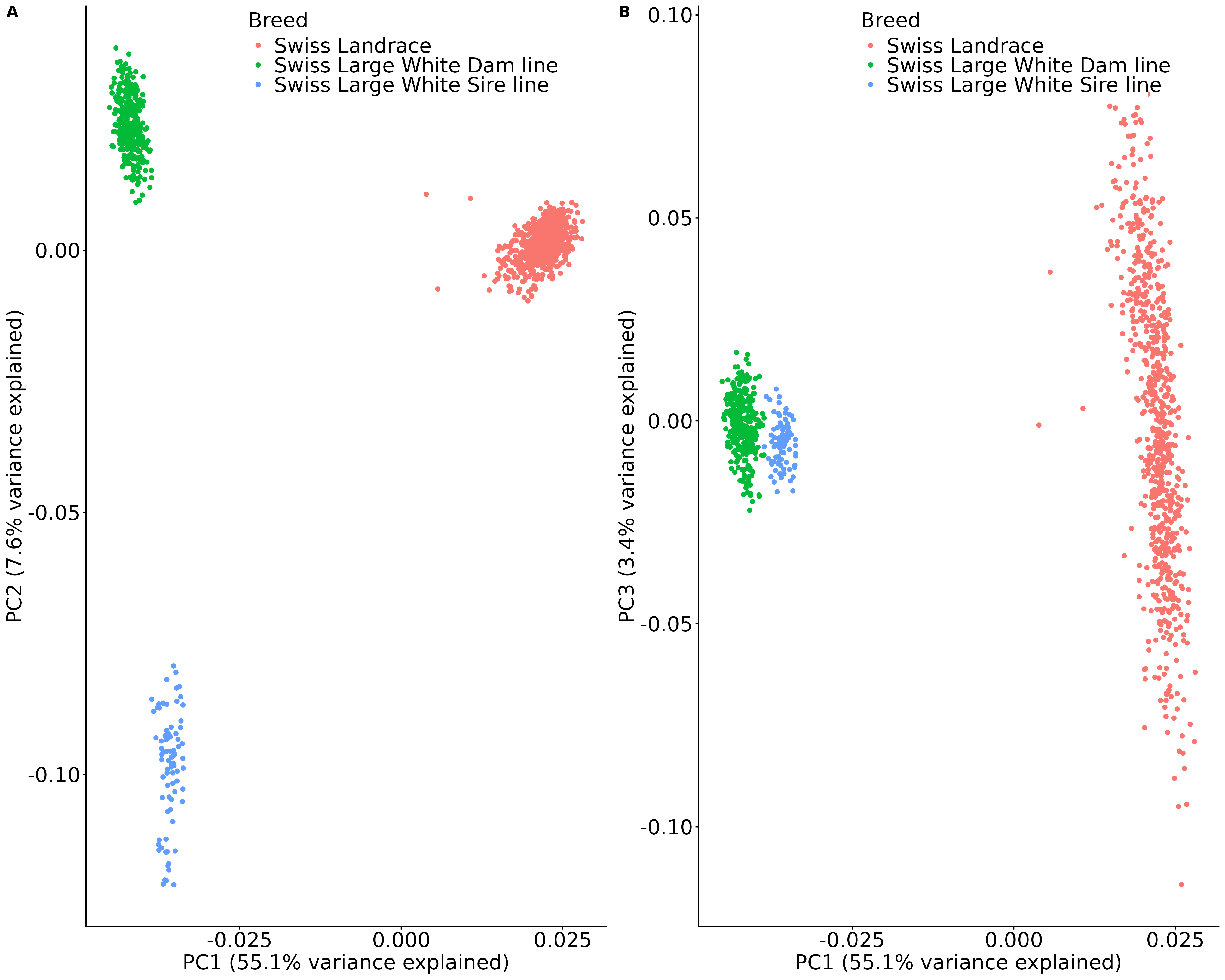

Supplement: Supplementary file 5 — Additional file5 (PNG 505 KB) [file 12711_2026_1040_MOESM5_ESM.png]

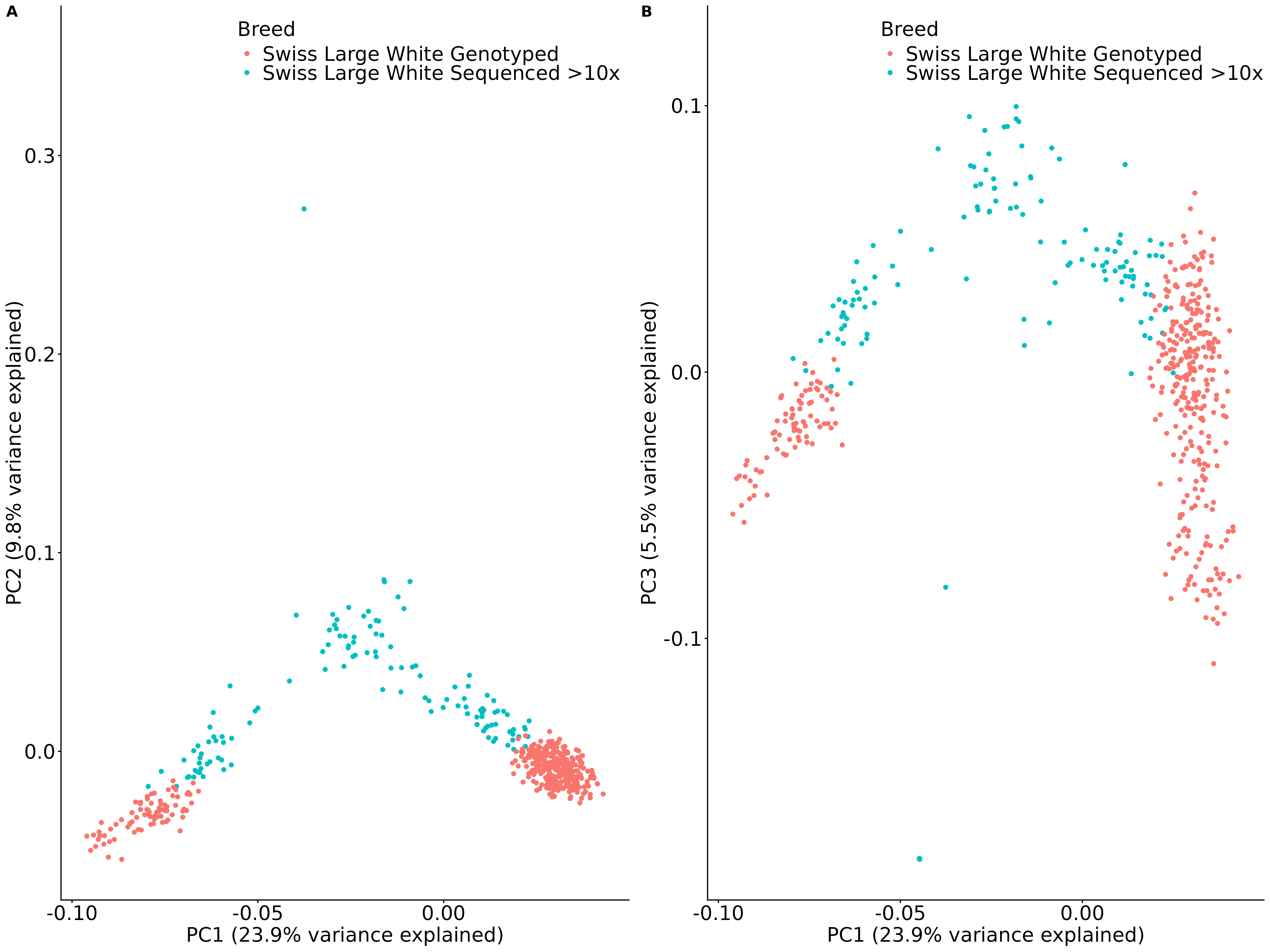

Supplement: Supplementary file 6 — Additional file6 (PNG 495 KB) [file 12711_2026_1040_MOESM6_ESM.png]

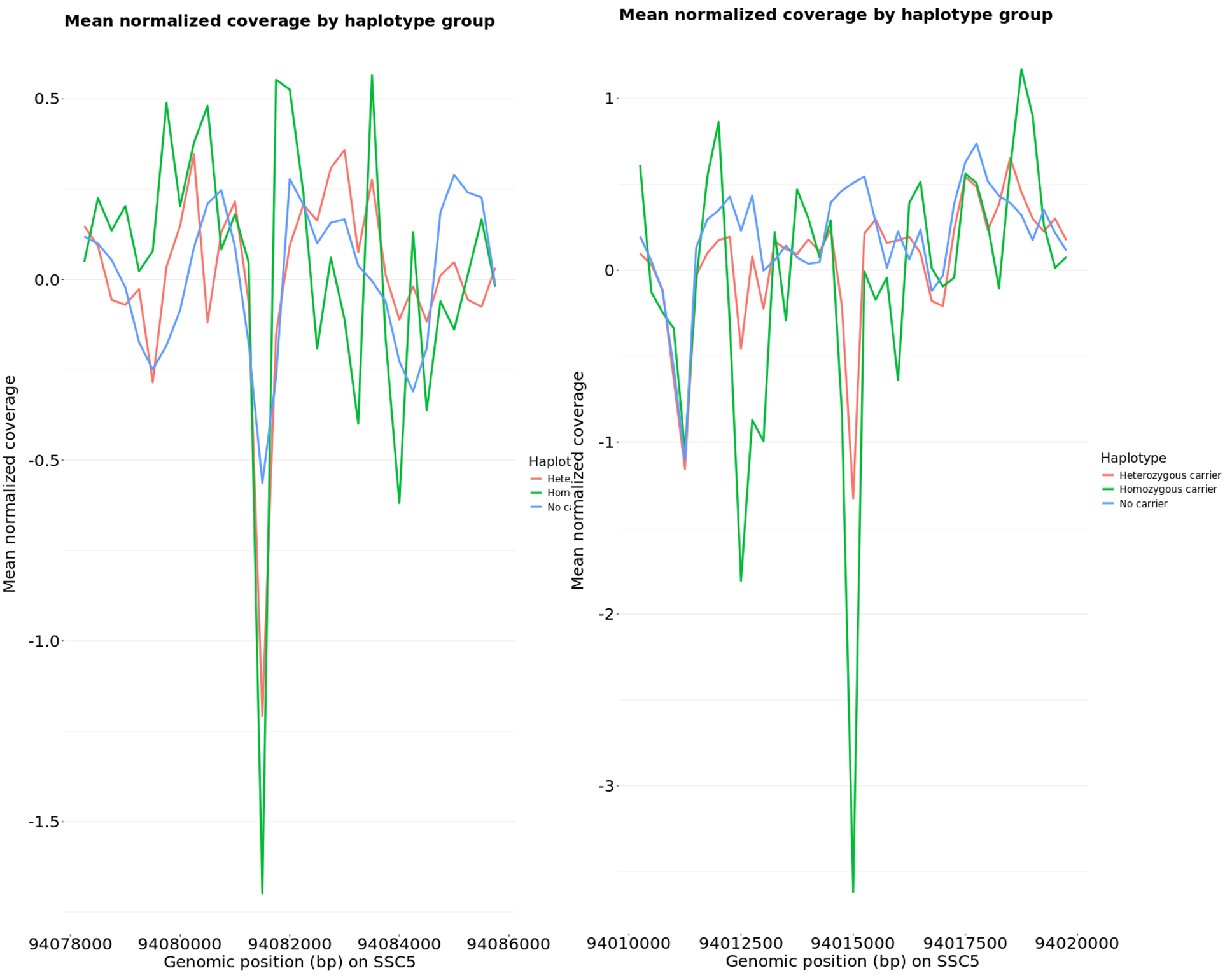

Supplement: Supplementary file 16 — Additional file16 (PNG 521 KB) [file 12711_2026_1040_MOESM16_ESM.png]

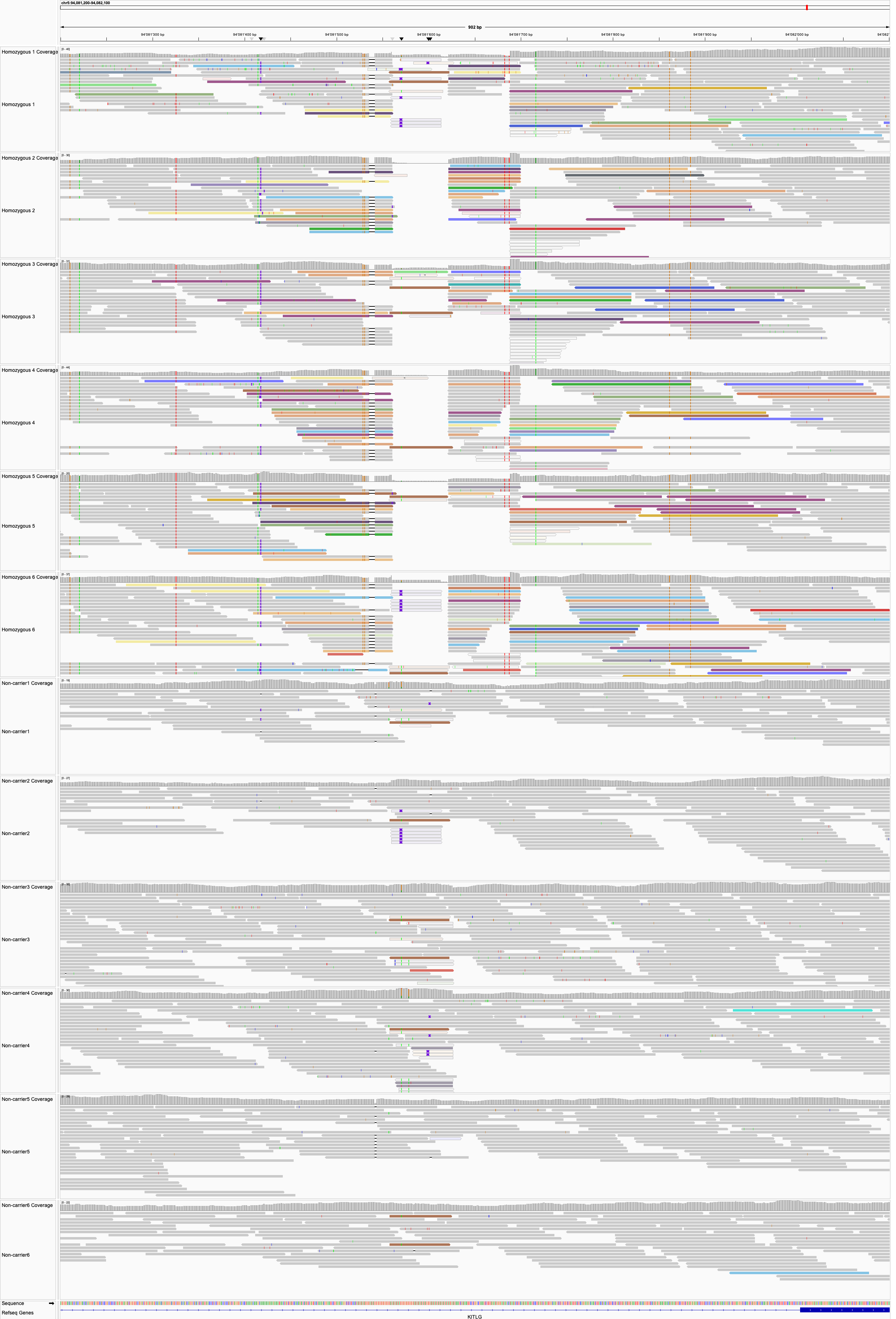

Supplement: Supplementary file 17 — Additional file17 (PNG 336 KB) [file 12711_2026_1040_MOESM17_ESM.png]

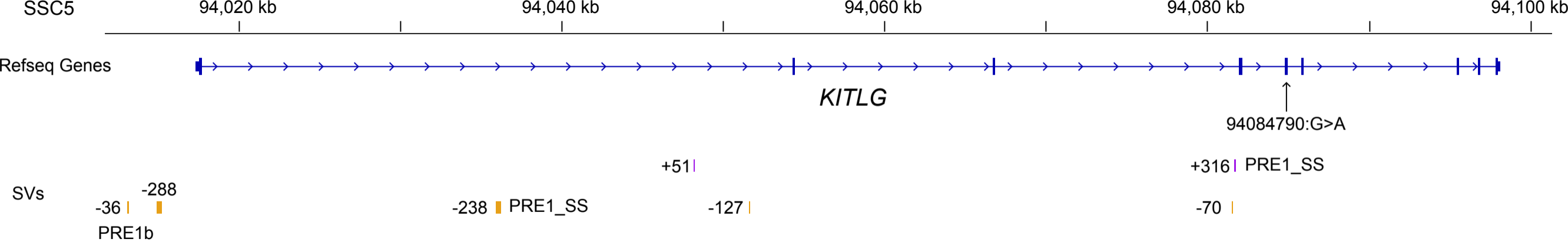

Supplement: Supplementary file 18 — Additional file18 (PDF 74 KB) [file 12711_2026_1040_MOESM18_ESM.pdf]
