# Supplementary material for: Genetic analysis of iris pigmentation in Swiss pig breeds identifies a missense KITLG variant as a potential causal factor for pale and heterochromatic irises
Source: Genet Sel Evol. 2026 Mar 25;58:22. doi: 10.1186/s12711-026-01040-1 (PMC13023151; doi:10.1186/s12711-026-01040-1)
Supplement: Supplementary file 20 — Additional file20 (DOCX 17 KB) [file 12711_2026_1040_MOESM20_ESM.docx]

**Additional files**

**Additional File 1 Table S1**

Format: .xlsx

Title: Prevalences of iris pigmentation in pigs reported by previous studies.

Description: Prevalences of iris pigmentation in pigs reported by previous studies. Not all studies used a similar approach for phenotyping: Nielsen and Lind [17] did not differentiate between heterochromia iridum and iridis, while Gelatt et al. [3] did not differentiate between pale and heterochromia.

**Additional File 2 Figure S1**

Format: .jpg

Title: Phenotyping scale for iris pigmentation, following Moscatelli et al. (2020)

Description: Phenotyping scale for iris pigmentation, following Moscatelli et al. (2020) [4]. In this study, medium and dark brown irises were grouped together in ‘dark’, as it was hard to distinguish both types in live pigs in a farm environment.

**Additional File 3 Figure S2**

Format: .jpg

Title: Histogram of production traits distribution for the Swiss Landrace population.

Description: Top left: Histogram of the age at recording phenotype. Top right: Histogram of life daily gain (g/d). Bottom left: Histogram of backfat thickness (mm). Bottom right: Histogram of loin muscle depth (mm).

**Additional File 4 Figure S3**

Format: .jpg

Title: Histogram of production traits distribution for the Swiss Large White population.

Description: Top left: Histogram of the age at recording phenotype. Top right: Histogram of life daily gain (g/d). Bottom left: Histogram of backfat thickness (mm). Bottom right: Histogram of loin muscle depth (mm).

**Additional File 5 Figure S4**

Format: .png

Title: Principal component (PC) analysis of the Swiss Landrace and Swiss Large White array-derived genotypes.

Description: A: PC1 versus PC2, showing different subpopulations. B: PC1 versus PC3, showing Swiss Large White subpopulations cluster together.

**Additional File 6 Figure S5**

Format: .png

Title: Principal component (PC) analysis of genotyped Swiss Large White pigs versus sequenced Swiss Large White pigs.

Description: Left: A versus PC2. B: PC1 versus PC3.

**Additional File 7 Table S2**

Format: .xlsx

Title: Overview of number of phenotyped pigs per farm and per breed

Description: Number of pigs with a specified iris pigmentation phenotype per farm and per breed. In total, there were 837 Swiss Landrace, 328 Swiss Large White, 20 crossbreds from Swiss Landrace and Swiss Large White, 11 Duroc and 4 Piétrain pigs phenotyped. LDR: Swiss Landrace; LWT: Swiss Large White; HYB: Hybrid sow crossbred from Swiss Landrace and Swiss Large White; DUR: Duroc; PIT: Piétrain

**Additional File 8 Table S3**

Format: .xlsx

Title: Overview of iris pigmentation phenotypes per sex

Description: Number of pigs with a specified iris pigmentation phenotype per sex and per breed for Swiss Landrace and Swiss Large White pigs. A chi-squared test of independence revealed significant differences in iris pigmentation phenotypes between sexes (χ² test, p = 0.0004). The largest differences between females and males were observed for heterochromia iridum (17.8% vs. 11.4%), light brown (16.6% vs. 23.9%), and pale irises (8.0% vs. 5.3%).

**Additional File 9 Table S4**

Format: .xlsx

Title: Prevalence of offspring iris pigmentation phenotypes versus maternal phenotype for Swiss Landrace.

Description: The number of phenotyped sows (N_sow_) and the number of phenotyped offspring (N_off_) per sow phenotype are given between brackets. Heterochromia iridis unilateralis and bilateralis were combined to have a relevant sample size.

**Additional File 10 Table S5**

Format: .xlsx

Title: Genetic parameters and heritability estimates for iris pigmentation in single trait 5-category threshold model.

Description: Main genetic parameters are shown for the single trait analysis for both the Swiss Landrace and the Swiss Large White.

**Additional File 11 Table S6**

Format: .xlsx

Title: Genetic parameters, heritability estimates and genetic correlations estimated for iris pigmentation and production traits in a bivariate threshold model with a 5-category trait and a linear trait.

Description: Main genetic parameters are shown for the bivariate analysis for both the Swiss Landrace and the Swiss Large White. Swiss Large White estimates were not shown in the manuscript due to a limited sample size.

**Additional File 12 Table S7**

Format: .xlsx

Title: GWAS results of most significant associations per 1Mb bin per scenario surpassing the suggestive threshold of P<10^-5^.

Description: GWAS results of most significant associations per 1Mb bin per scenario surpassing the suggestive threshold of P<10^-5^ for the three studied populations (‘breed’; Swiss Landrace, Swiss Large White and combined) and GWAS methods (haplotypes or imputed sequences) for different scenarios as explained in Table 3 (‘trait’).

**Additional File 13 Table S8**

Format: .xlsx

Title: GWAS results of all variants surpassing the suggestive threshold of P<10^-5^.

Description: GWAS results of all variants surpassing the suggestive threshold of P<10^-5^ for the three studied populations (‘breed’; Swiss Landrace, Swiss Large White and combined) and GWAS methods (haplotypes or imputed sequences) for different scenarios as explained in Table 1 (‘trait’).

**Additional File 14 Table S9**

Format: .xlsx

Title: VEP annotation of 14,835 iris pigmentation-associated variants

Description: This table contains the Ensembl Variant Effect Predictor (VEP) annotations for 14,835 SNPs and indels that surpassed the genome-wide association threshold (P < 5×10⁻^8^) in at least one iris pigmentation GWAS comparison. Each variant is annotated with its predicted functional consequence, including coding impact (e.g., synonymous, missense), location (e.g., intronic, intergenic), and overlap with known genes or regulatory features.

**Additional File 15 Table S10**

Format: .xlsx

Title: Genotype distribution of missense variant 5_94084790_G>A across pig populations.

Description: This table contains genotype distribution of the missense variant 5_94084790_G>A from 1239 pigs across 43 pig populations. Data were downloaded via: <https://quantgenet.msu.edu/swim/statistics.php>. European commercial breeds and European wild boar are almost fixed for the reference (G) allele, whereas many Asian breeds are fixed or nearly fixed for the alternate (A) allele.

**Additional File 16 Figure S6**

Format: .png

Title: Mean normalized coverage per haplotype associated with iris pigmentation in Swiss Large White pigs

Description: Mean normalized coverage was estimated via Mosdepth per 250 bp interval based on short-read sequence data of 120 Large White pigs with at least 10x coverage. From this cohort, 6 homozygous carriers were identified for a haplotype associated with iris pigmentation, 39 heterozygous carriers and 75 non-carriers. Left: Mean normalized coverage distance within the *KITLG* gene near exon 2. Right: Mean normalized coverage distance near a 288bp deletion at Chr5:94,014,953, just 2.4 Kb upstream of the first *KITLG* exon (Additional File 19 Table S11). This deletion matched the Pre0_SS element, which is also a SINE element of the PRE1 family.

**Additional File 17 Figure S7**

Format: .png

Title: Integrative Genomics Viewer (IGV) plot showing a potential insertion in the six homozygous haplotype carriers (top tracks) compared to six randomly selected non-carriers (bottom tracks).

Description: This IGV screenshot shows short-read alignments and genome coverage across chromosome 5 (Chr5:94,081,200–94,082,100), centered on a suspected insertion site at approximately Chr5:94,081,500. Each horizontal track represents sequencing reads from an individual. The upper six tracks correspond to homozygous haplotype carriers, and the lower six tracks show randomly selected non-carriers. Gray bars indicate reads that align continuously to the reference genome. Colored segments within reads mark mismatches or clipped regions, which may reflect structural variation. The vertical colored lines correspond to base mismatches (e.g., A=green, T=red, G=orange, C=blue). Regions with reduced or missing read coverage (gaps or white spaces) and the presence of soft-clipped reads (colored ends) near Chr5:94,081,500 suggest an insertion event in the carriers. The coverage track above each group summarizes read depth, where dips indicate lower coverage.

**Additional File 18 Figure S8**

Format: .png

Title: Insertions and deletions detected near the *KITLG* gene in LD (r^2^>0.8) with missense variant 5_94084790_G>A.

Description: For the 18 pigs with HiFi long-read sequence information, several deletions and insertions were found in LD (r^2^>0.8) with missense variant 5_94084790_G>A.

**Additional File 19 Table S11**

Format: .xlsx

Title: Insertions and deletions detected near the *KITLG* gene in LD (r^2^>0.8) with missense variant 5_94084790_G>A.

Description: Structural variants in high linkage disequilibrium (LD; r2>0.8) with the missense variant 5_94084790_G>A in the 92-96Mb region near the KITLG on SSC5.
